# Climate Change and Human Pressure: Assessing the Vulnerability of Snow Leopard ( *Panthera uncia* ) Habitat Integrated With Prey Distribution on the Qinghai‐Tibet Plateau

**DOI:** 10.1002/ece3.71232

**Published:** 2025-04-04

**Authors:** Yu Zhang, Yunchuan Dai, Jia Li, Wei Cong, Yuguang Zhang, Xiuqing Nie, Qiong Wu, Yadong Xue

**Affiliations:** ^1^ Ecology and Nature Conservation Institute Chinese Academy of Forestry Beijing China; ^2^ Key Laboratory of Biodiversity Conservation of National Forestry and Grassland Administration Beijing China; ^3^ Institute for Ecology and Environmental Resources Chongqing Academy of Social Sciences Chongqing China; ^4^ Institute of Ecological Conservation and Restoration Chinese Academy of Forestry Beijing China; ^5^ Research Institute of Forestry Chinese Academy of Forestry Beijing China

**Keywords:** climate change, cross‐provincial conservation, dietary analysis, habitat assessment, human‐wildlife conflicts

## Abstract

Climate change is significantly altering the distribution of large carnivores and their primary prey species, with particular emphasis on the changing prey distribution in high‐altitude regions. The Qinghai‐Tibet Plateau, known for its rich biodiversity, is highly sensitive to climate change, affecting the habitats of snow leopards ( *Panthera uncia* ) and blue sheep ( *Pseudois nayaur* ). Our study identified blue sheep as the primary prey of snow leopards through metagenomic analysis and used bioclimatic data and Land Use/Cover Change (LUCC) information to model habitat suitability under three climate scenarios (RCP 2.6, RCP 4.5, and RCP 8.5). Projections showed that under RCP 4.5 and RCP 8.5, snow leopard habitats will decrease by 13.0% and 23.4%, while blue sheep habitats will decrease by 38.3% and 49.7%, respectively. These habitats are expected to shift to higher altitudes, with snow leopards experiencing a more significant shift. Based on these findings, we recommend adjusting protected area boundaries for S1 (Ideal distribution range), establishing ecological corridors for S2 (stepping stone), and implementing targeted measures to mitigate human‐wildlife conflicts in S3 (potential conflict area). To protect these species, international efforts to reduce carbon emissions, cross‐administrative cooperation, and community‐based conservation strategies are essential.

## Graphical abstract

Our study revealed the changing patterns of snow leopards and their main prey distribution areas under different climate change scenarios.

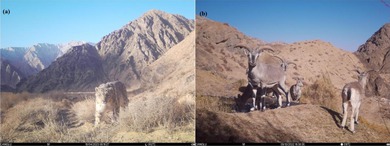

## 1 Introduction

Understanding how animals adapt to fluctuating environments is crucial for comprehending species responses to ecological constraints. This becomes especially urgent in the context of climate change, which can alter both biotic and abiotic conditions preferred by animals (Beumer et al. 2020). The spatial distribution of carnivores is influenced by factors such as terrain, habitat quality, prey abundance, and human activities (Liu et al. 2021). Threats like overgrazing, human activities, and climate change have led to population declines and habitat degradation among carnivores (Ripple et al. 2014), potentially impacting interspecies interactions. As carnivore and prey populations fluctuate, interspecific competition intensifies. Addressing niche strategies for carnivores to alleviate competitive pressures and understanding interspecific interactions crucial for maintaining multi‐species coexistence are pressing issues in biodiversity conservation and animal ecology.

The snow leopard ( *Panthera uncia* ) has a broad distribution across 12 countries: Afghanistan, Bhutan, China, India, Kazakhstan, Kyrgyzstan, Mongolia, Nepal, Pakistan, Russia, Tajikistan, and Uzbekistan (McCarthy et al. 2017). As a keystone species in the mountainous ecosystems of Central Asia, the survival status of the snow leopard is indicative of the overall health of these environments (Lyngdoh et al. 2014). Recognized as both the apex predator and a flagship species (McCarthy et al. 2016), it plays a crucial role in maintaining the stability of food webs and ecosystem balance across Central Asia and the Qinghai‐Tibet Plateau (Cardillo et al. 2004; Li, McCarthy et al. 2016; Li, Yu et al. 2016). Due to their elusive behavior and preference for remote mountainous habitats, gathering sufficient data across their range of countries remains challenging, hindering the formulation of detailed conservation plans (Mishra 2017; Liu et al. 2019). In 2017, the IUCN reclassified the snow leopard from Endangered to Vulnerable on the Red List, a decision that has been met with skepticism by some researchers. They argue that despite decades of global research efforts, credible population density estimates are scarce, and current assessments are based on surveys covering only a fraction of the snow leopard's extensive habitat (Mishra 2017; Ale and Mishra 2018). Therefore, there remains a critical need for further investigation and research into the distribution and habitat requirements of the snow leopard.

The blue sheep ( *Pseudois nayaur* ) occupies a distinct ecological niche compared to the snow leopard. While the snow leopard is a carnivorous apex predator that regulates the population of herbivores and maintains ecosystem balance, the blue sheep is a herbivorous prey species that significantly influences the vegetation dynamics and landscape structure. The blue sheep inhabits a vast range spanning from the Qinghai‐Tibet Plateau to the Himalayas, extending from southwest Yunnan in China through northeast India, Bhutan, Sikkim, Nepal, and northwest India, reaching as far as the Shimshal Valley in the Karakoram Mountains of northern Pakistan. Its northeastern limit extends to the Helan Mountains in China, typically found at altitudes ranging from 2500 to 5500 m (Schaller 1977). According to Harris (2014), the total population of blue sheep is estimated to be between 47,000 and 414,000 individuals, indicating its significant abundance across most areas of the Qinghai‐Tibet Plateau. The IUCN Red List categorizes the blue sheep as Least Concern (Harris 2014). Although the snow leopard and blue sheep share overlapping habitats, their ecological roles are fundamentally different. The habitat of the blue sheep overlaps extensively with that of the snow leopard on the Qinghai‐Tibet Plateau. This cohabitation is significant because snow leopards rely heavily on blue sheep as a primary prey species in their ecosystem. Understanding the distribution and dynamics of blue sheep populations is therefore crucial for assessing the ecological health and conservation status of snow leopards in these high‐altitude regions.

Snow leopards inhabit high‐altitude regions characterized by challenging environmental conditions, where the availability of prey, in terms of both population density and diversity, is relatively lower compared to carnivores at lower altitudes (Oli et al. 1993; Křenová et al. 2022; Koju et al. 2023; Lovari et al. 2024). Studies have shown that snow leopards in Nepal primarily rely on prey species such as blue sheep ( *Pseudois nayaur* ), Himalayan musk deer ( *Moschus leucogaster* ), and livestock, with significant seasonal variations in prey availability (Koju et al. 2023). These factors contribute to the snow leopard's ability to adapt its diet based on prey abundance in different seasons (Křenová et al. 2022). Among their primary prey species, the Siberian ibex ( *Capra sibirica* ) is also widely documented (Nyhus et al. 2016), covering almost the entirety of the snow leopard's range. Lyngdoh et al. (2014) identified several key wild prey species for snow leopards across their distribution, including blue sheep, Siberian ibex, Himalayan tahr ( *Hemitragus jemlahicus* ), argali ( *Ovis ammon* ), and Himalayan marmot ( *Marmota himalayana* ). Dietary composition varies significantly by region: in Nepal, snow leopards predominantly prey on blue sheep and Himalayan tahr (Aryal et al. 2014; Thapa et al. 2021), while in Pakistan, approximately 70% of their diet consists of domestic animals, with blue sheep and markhor ( *Capra falconeri* ) comprising the remainder (Anwar et al. 2011). In India, blue sheep are the primary prey (Chundawat and Rawat 1994). In the Gobi region of Mongolia, snow leopards primarily feed on argali and Siberian ibex due to limited prey species (Wasim et al. 2012), whereas in Gansu, Qinghai, and Xinjiang provinces of China, their diet includes blue sheep and marmots (Lu et al. 2019). Generally, snow leopards exhibit significant spatial overlap with blue sheep, particularly on the Qinghai‐Tibet Plateau (Chi et al. 2019; Wang et al. 2024). Understanding the dynamics of prey availability and dietary preferences across these diverse habitats is essential for effective conservation strategies aimed at safeguarding this iconic species. These findings underscore the importance of targeted conservation efforts to ensure sustainable prey populations in snow leopard habitats.

Climate change is widely acknowledged as a significant driver of biodiversity loss, with anticipated impacts on species distribution, ecological relationships, and habitat suitability (Aryal et al. 2014; Xue et al. 2021). Li, McCarthy et al. 2016; Li, Yu et al. 2016 argued that global warming indirectly exacerbates the fragmentation of snow leopard habitats, particularly affecting high‐altitude vertical spaces. The shrinking alpine zones are expected to reduce snow leopard habitat in the Himalayas by up to 30% (Forrest et al. 2012). Future climate change is projected to alter the spatial dynamics between snow leopards and their primary prey, such as blue sheep, potentially decreasing habitat overlap and increasing ecological mismatches (Aryal et al. 2014). Given that snow leopards primarily inhabit mountainous regions, these changes are likely to have profound implications for their survival and ecological function (Bhatti et al. 2022). Researchers have extensively studied the distribution of snow leopard habitats in the context of both land use change and climate change (Aryal et al. 2014; Li, McCarthy et al. 2016; Li, Yu et al. 2016; Li et al. 2021; Li et al. 2022; Ue et al. 2024). Understanding these dynamics is crucial for developing effective conservation strategies that mitigate the impacts of climate change on snow leopards and their ecosystems.

**FIGURE 1:**
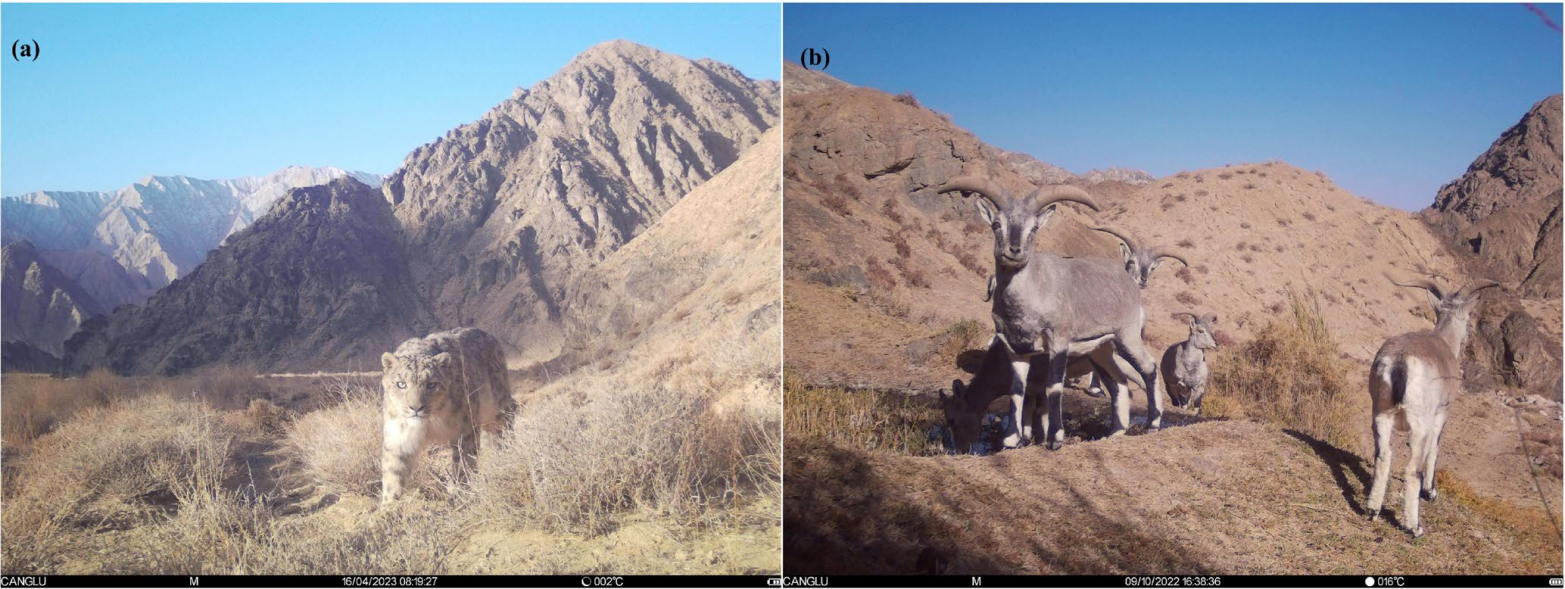
The snow leopard ( *Panthera uncia* ; a) and blue sheep ( *Pseudois nayaur* ; b) captured by camera trapping on the Qinghai‐Tibet Plateau.

The snow leopard (Figure 1a) serves as a flagship species for conservation efforts on the Qinghai‐Tibet Plateau, as this area harbors a significant portion of the world's snow leopard habitat. Considering the complex terrain and inconvenient transportation in the region, conducting comprehensive field surveys is quite challenging. Modeling the spatial distribution of snow leopards and their primary prey, the blue sheep (Figure 1b), is crucial for understanding habitat preferences and guiding effective conservation strategies. In this study, we utilized climate variables and Land Use/Cover Change (LUCC) data to evaluate the habitat suitability for snow leopards. Preliminary metagenomic analyses identified the blue sheep as the primary food source for snow leopards. Following this, we delineated the potential distribution ranges of both species to gain a deeper understanding of their habitat requirements. These findings are essential for developing informed conservation strategies aimed at preserving snow leopards in their natural environments amidst ongoing environmental changes. Our research makes a significant contribution to the understanding of snow leopard ecology and provides valuable insights for targeted conservation initiatives across the Qinghai‐Tibet Plateau.

## 2 Material and Methods

### 2.1 Study Area

**FIGURE 2:**
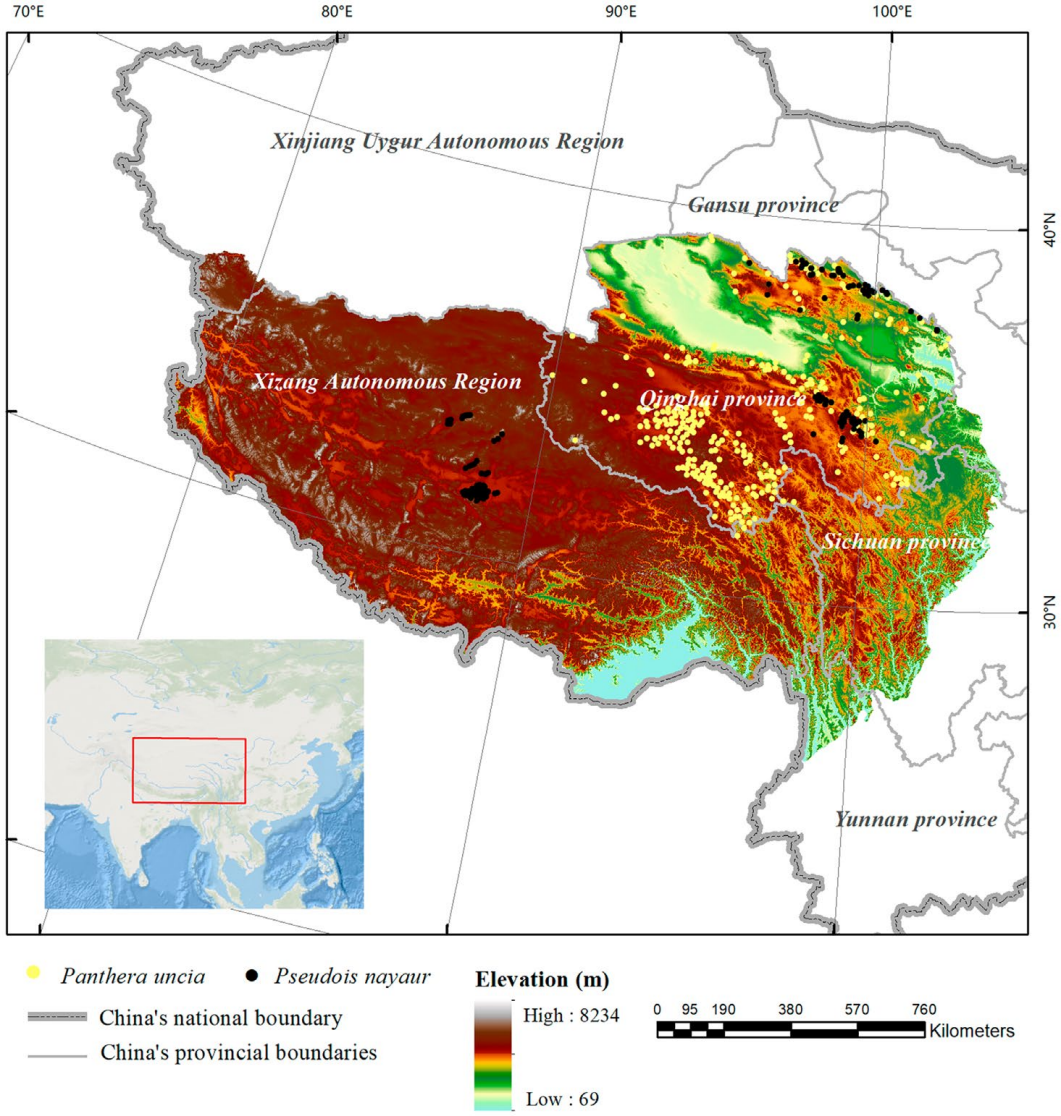
Location on the Qinghai‐Tibet Plateau.

The Qinghai‐Tibet Plateau (Figure 2), often referred to as the “Roof of the World,” represents the highest and largest plateau on Earth, encompassing an area of approximately 2.5 million km^2^. The Qinghai‐Tibet Plateau includes the entire Xizang Autonomous Region, as well as parts of Qinghai, Xinjiang, Gansu, Sichuan, and Yunnan provinces of China. It also covers part or all of Bhutan, Nepal, India, Pakistan, Afghanistan, Tajikistan, and Kyrgyzstan. The Qinghai‐Tibet Plateau is distinguished by significant geological activities that contribute to its unique ecological diversity and dynamism. As one of the 36 global biodiversity hotspots, it encompasses 60 out of the 200 global ecoregions (Chettri et al. 2008), harboring abundant biodiversity. This region serves as a critical habitat for numerous rare and endangered species, including large mammals such as the snow leopard, Tibetan brown bears ( *Ursus arctos pruinosus* ), Himalayan brown bears (*U. a. isabellinus*), and blue sheep (Khan and Baig 2020). These species have adapted to the unique high‐altitude ecosystems of the Qinghai‐Tibet Plateau, intricately linked with its distinctive terrain and climate, making them indispensable components of the region's ecosystem.

### 2.2 Data Collection

#### 2.2.1 Scat Sampling and Dietary Analysis

A total of 360 high‐quality scats of snow leopard were collected in four areas from July 2018 to June 2021, including Qilian mountain (266), Hoh Xil (2), Dulan county (77), and Yushu county (15) in China. DNA was extracted using the QIAamp Fast DNA Stool Mini Kit (QIAGEN, Germany) following standard protocols. The MT‐RNR1 (12S) and COX1 (cytochrome c oxidase subunit I) gene segments were amplified using 12SV5‐F/R primer and COX1 primers, respectively (Hacker et al. 2021; Riaz et al. 2011). PCR reaction conditions followed the methods described in Hacker et al. (Hacker et al. 2021; Cong et al. 2023). The resulting library was sequenced on an Illumina NovaSeq platform, and 250 bp paired‐end reads were generated (Guangdong Magigene Biotechnology Co. Ltd. Guangzhou, China).

Analysis methods of sequence data are described in Hacker et al. (2021, 2022). To determine the prey consumed by snow leopard, Genomics Workbench v12.0 (QIAGEN, Denmark) was used by mapping sequence reads to reference sequences of possible prey. Reference sequences were downloaded from GenBank and BOLD (Barcode of Life Data Systems) with representative haplotypes compiled into one fasta file. Raw reads were required to have at least 98% similarity across at least 90% of the reference sequence for mapping (Hacker et al. 2021). Prey identification were made based on the reference taxa with the highest number of reads mapped and the fewest mismatches. Samples were considered to have more than one prey item if the amount of reads for each identified prey item accounted for a large portion of the total read count. Samples in which species could not be identified were analyzed to ensure the reference file was not incomplete by using the de novo assembly tool in CLC, then blasting the resulting contig sequence with the nucleotide databases in NCBI (https://blast.ncbi.nlm.nih.gov/Blast.cgi). Samples that did not map reads to any prey species were categorized as “Unknown” As an additional precaution, the geographic range of prey species was researched using the IUCN Red List (https://www.iucnredlist.org/) to ensure that it overlapped with the study site. Dietary data were summarized by the frequency of occurrence of prey species in scats observed.

#### 2.2.2 Collection of Species Occurrence

We collected a total of 487 GPS coordinates for snow leopards and 325 for blue sheep. Among these, 186 coordinates were obtained from ground surveys conducted between 2017 and 2023, documenting occurrences of snow leopards and blue sheep, including sightings of individuals, feces, tracks, hair, and feeding signs. One hundred ninety‐six coordinates were derived from infrared camera traps spanning from 2016 to 2023. Additionally, 430 coordinates were sourced from the published literature (Hameed et al. 2020) and the non‐governmental organizations (Plateau Nature Conservancy; Wildlife Conservation Society China Program) (Figure 2). To mitigate spatial autocorrelation, one point was randomly selected within each 10 km^2^ grid cell for modeling analysis (Dai, Peng et al. 2021; Dai, Hacker et al. 2021).

### 2.3 Biovariables and Environmental Data

Nineteen bioclimatic variables for current climatic conditions (average for 1950–2000) were sourced from the WorldClim 1.4 database at a resolution of 1 km. To predict the future (average for 2061–2080, hereafter referred to as the 2070s) distributions of snow leopard and blue sheep, we selected three widely used General Circulation Models (GCMs) in Asia (Ye et al. 2018; Yu et al. 2019). Each GCM was applied with three emission scenarios (Representative Concentration Pathways: RCP 2.6, RCP 4.5, and RCP 8.5) to reflect varying greenhouse gas concentrations over the coming decades (available at https://www.worldclim.org/). The current (2010) and future (2070s) LUCC data were obtained from the Finer Resolution Observation and Monitoring‐Global Land Cover (30 m resolution; available at http://data.ess.tsinghua.edu.cn/data/Simulation/; Li, McCarthy et al. 2016; Li, Yu et al. 2016). To align LUCC data with climate data, we selected the years 2010 and 2070s, and the RCP 2.6, RCP 4.5, and RCP 8.5 as representatives of future LUCC scenarios. Additional environmental variables include Digital Elevation Model (DEM) data at 30 m resolution (available at http://www.gscloud.cn/) and Human Influence Index (HII) data at 1 km resolution (available at http://sedac.ciesin.columbia.edu/).

The selected bioclimatic variables, including temperature and precipitation, are highly relevant to the ecology of both snow leopards and blue sheep. Snow leopards are adapted to cold, high‐altitude environments, and their distribution depends on prey availability and climatic conditions. Blue sheep, as a key prey species for snow leopards, also rely on these variables, as they inhabit similar high‐altitude regions. Therefore, these bioclimatic variables were chosen for their direct influence on the habitat suitability of both species. Due to the unavailability of future HII and DEM data, we referred to previous studies and assumed that these two variables remain unchanged in the predictive models (Stanton et al. 2012). Finally, we standardized the spatial coordinates, spatial resolution, and extent of each variable in ArcGIS 10.6 (ESRI Inc., Redlands, CA, USA).

### 2.4 Habitat Suitability Modeling

The MaxEnt model (Maximum Entropy Model) is a statistical model used to assess species habitat suitability, based on the principle of maximizing entropy to estimate the most likely species distribution (Phillips and Dudík 2008; Phillips et al. 2006). It is a machine learning method employed for species distribution assessment, leveraging the principle of maximum entropy, which selects the probability distribution with maximum entropy under a given set of constraints as the most reasonable model. In ecological and biodiversity studies, the MaxEnt model is widely utilized to predict species suitable habitats, particularly when species have incomplete records or sparse distribution data. Typically, input variables for the MaxEnt model include environmental data such as terrain, climate, LUCC, and human activities. These variables describe the ecological characteristics that influence species habitat selection. The model analyzes environmental conditions at known species occurrence points to learn the relationships between species and environmental variables and subsequently predicts potential suitable habitats in unknown areas (Phillips et al. 2006). The MaxEnt model offers several advantages, including its capability to handle high‐dimensional environmental data and relatively small species occurrence datasets. Furthermore, it can provide probability distributions of species presence under specific environmental conditions, rather than just single‐point predictions.

The parameters for the MaxEnt model were configured as follows: a 25% random test percentage and a regularization multiplier of 1. Fifteen replicates were conducted, and cross‐validation was performed to assess model robustness. The remaining parameters were chosen based on previous studies (Dai, Peng et al. 2021; Dai, Hacker et al. 2021; Phillips et al. 2006). The performance of the MaxEnt model was assessed using the area under the receiver operating characteristic curve (AUC). AUC serves as an independent metric to evaluate the accuracy of model predictions, with values ranging from 0 to 1 (Phillips et al. 2006). Higher values, closer to 1, signify a more reliable model. We selected the average logistic threshold value of Maximum Training Sensitivity Plus Specificity (MTSPS) to identify suitable habitats because it optimizes the balance between sensitivity (correctly identifying suitable habitats) and specificity (correctly identifying unsuitable habitats). This threshold method is widely used in the MaxEnt model (Dai, Peng et al. 2021; Dai, Hacker et al. 2021). Grids with probability values exceeding this threshold were classified as suitable habitats.

### 2.5 Assessing Habitat Suitability Across Three Scenarios

To scientifically assess the habitat suitability of snow leopards, we proposed three habitat scenario hypotheses based on the distributions of snow leopards and their primary prey.

#### 2.5.1 Scenario 1 (Ideal Distribution Range; S1)

Overlap of prey and habitat distribution. This scenario represents the ideal state where snow leopard habitats highly coincide with the distribution areas of their primary prey, the blue sheep, providing the most suitable conditions for their survival.

#### 2.5.2 Scenario 2 (Stepping Stone; S2)

Presence of prey distribution but absence of habitat distribution. In this case, areas with blue sheep distribution but lacking suitable habitats for snow leopards were considered as “stepping stones,” indicating potential adaptive challenges or inadequacies for snow leopards in these regions.

#### 2.5.3 Scenario 3 (Potential Conflict Area; S3)

Presence of habitat distribution but absence of prey distribution. Here, habitats exist, but the distribution of blue sheep is limited or absent, suggesting potential conflict zones over resources that may threaten the survival of snow leopards.

Through the simulation and analysis of these scenarios, we comprehensively assess the complexity of snow leopard habitats and their relationship with food resources.

## 3 Results

### 3.1 Snow Leopard Food Preferences

Prey items were identified in 341 (94.7%) scats of snow leopard, and the remaining 19 (5.3%) samples did not map reads to any prey species. Blue sheep, Himalayan marmot, and plateau pika constituted 78.8% of the snow leopard's diet (*N* = 286), 54.3%, 14.6%, and 9.9%, respectively. In most regions, blue sheep dominated the snow leopard's most important diet components, with the highest percentage in Yushu county (80.0%), followed by Dulan county (65.7%) and Qilian Mountains (50.4%). In Hoh Xil, there was a small number of scats, with a consistent proportion of blue sheep, domestic yak, and Himalayan marmot. Livestock were present in 9.6% of snow leopard diet counts (including domestic goat, sheep, yak, and horse) (Table 1). With the exception of Yushu, where no livestock were discerned, the domestic yak was the dominant livestock in the snow leopard's diet.

**TABLE 1:** The diet composition in snow leopard scat based on total occurrences of each prey species and the percentage of prey species.

Prey item	All	Qilian mountain	Dulan	Yushu	Hoh Xil
Domestic Horse	1 (0.3%)	1 (0.4%)			
Domestic Yak	27 (7.4%)	22 (8%)	4 (5.7%)		1 (33.3%)
Domestic Sheep	2 (0.6%)	2 (0.7%)			
Domestic Goat	5 (1.4%)	5 (1.8%)			
Himalayan Marmot	53 (14.6%)	42 (15.3%)	9 (12.9%)	1 (6.7%)	1 (33.3%)
Blue Sheep	197 (54.3%)	138 (50.4%)	46 (65.7%)	12 (80%)	1 (33.3%)
Wild Yak	11 (30%)	11 (4%)			
Himalayan Snowcock	4 (1.1%)	4 (1.5%)			
Plateau Pika	36 (9.9%)	27 (9.9%)	7 (10%)	2 (13.3%)	
Irenes Mountain Vole	3 (0.8%)	3 (1.1%)			
Wooly Hare	12 (3.3%)	11 (4%)	1 (1.4%)		
Eurasian Badger	1 (0.3%)	1 (0.4%)			
Musk Deer	2 (0.6%)	2 (0.7%)			
Tibetan Fox	1 (0.3%)	1 (0.4%)			
Red Fox	2 (0.6%)		2 (2.9%)		
*Buteo* sp.	1 (0.3%)	1 (0.4%)			
Eurasian Eagle Owl	1 (0.3%)	1 (0.4%)			
*Eospalax* sp.	1 (0.3%)		1 (1.4%)		
Qinghai Vole	2 (0.6%)	1 (0.4%)			
Pallas Cat	1 (0.3%)	1 (0.4%)			
Scats Number	360	266	77	15	2
Prey items identified scats number	341	256	68	15	2
Total Occurrences	363	274	70	15	3
Species	20	18	7	3	3
% Livestock Occurrences	9.6%	10.9%	5.7%	0.0%	33.3%
% Wild Species Occurrences	90.4%	89.1%	94.3%	100.0%	66.7%

### 3.2 Model Performance

**FIGURE 3:**
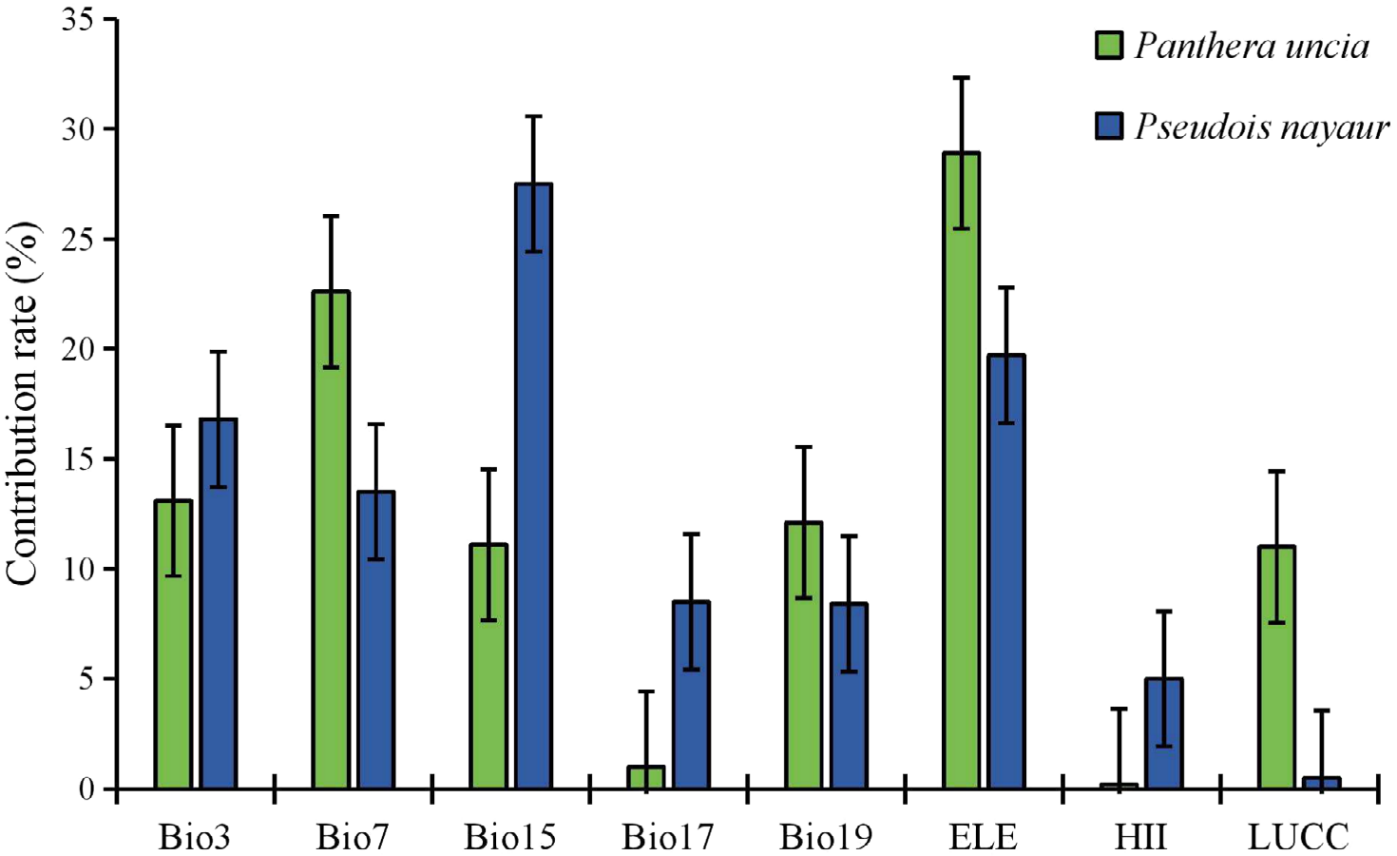
Importance of climate and environmental variables for snow leopard ( *Panthera uncia* ) and blue sheep ( *Pseudois nayaur* ).

In the MaxEnt model, 342 snow leopard occurrence points and 154 blue sheep occurrence points were used to develop Species Distribution Models (SDMs). Based on multicollinearity testing, eight variables were selected as input factors for these species. Climate and environmental factors exerted significant influences on the distributions of both species across three scenarios, with species‐specific impacts. Overall, altitude and climate variables made substantial contributions to the models. For snow leopards, elevation (ELE), Temperature Annual Range (Bio7), and Temperature Constancy (Bio3) contributed 28.9%, 22.6%, and 13.1% of the total contribution, respectively. For blue sheep, Precipitation Seasonality (Bio15), Altitude (ELE), and Temperature Constancy (Bio3) made significant contributions, averaging 27.5%, 19.7%, and 16.8%, respectively (Figure 3). Cross‐validation indicated excellent model performance for both snow leopards and blue sheep, with average testing AUC values of 0.905 and 0.970, respectively.

### 3.3 Projected Distributions of Snow Leopard and Blue Sheep

**FIGURE 4:**
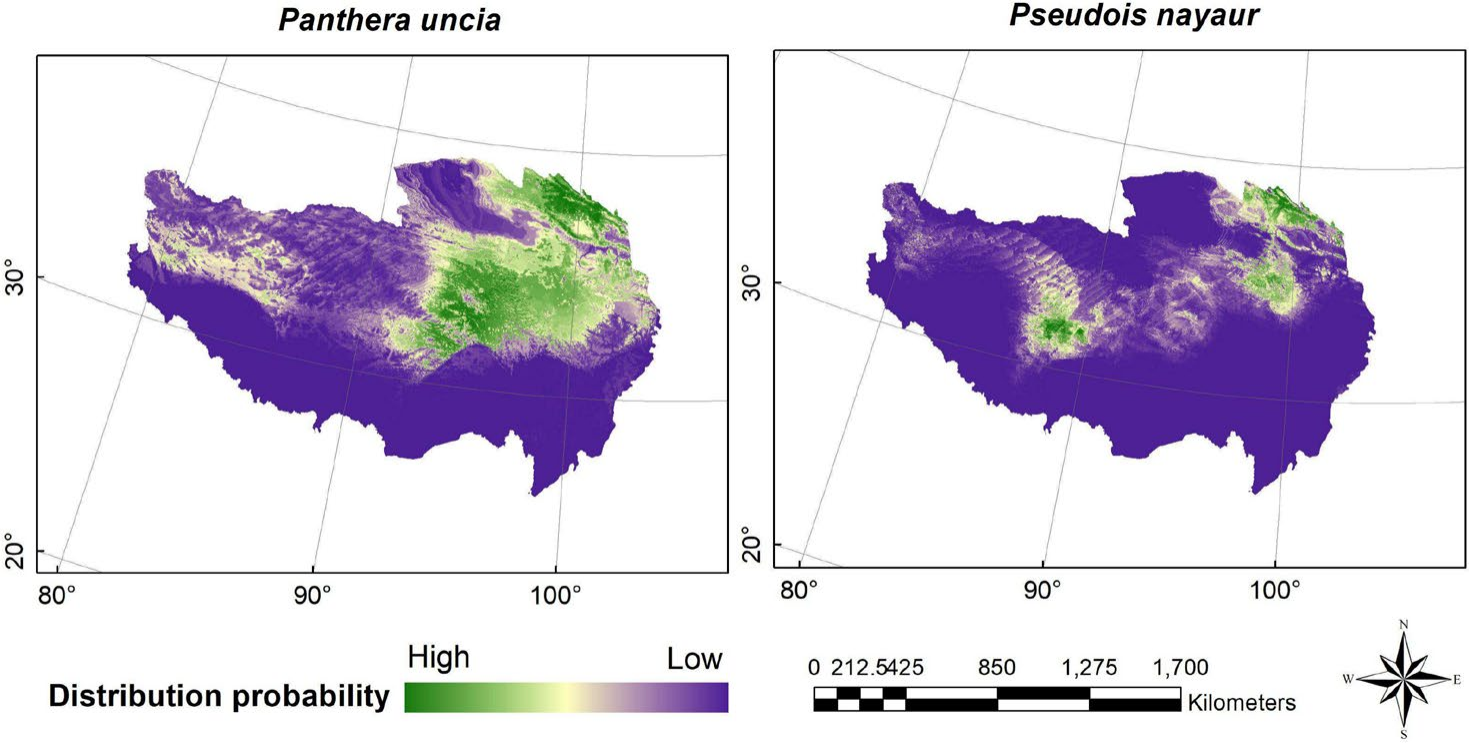
The current distribution probability of snow leopard ( *Panthera uncia* ) and blue sheep ( *Pseudois nayaur* ) under current and future climate scenarios and land use changes on the Qinghai‐Tibet Plateau.

**FIGURE 5:**
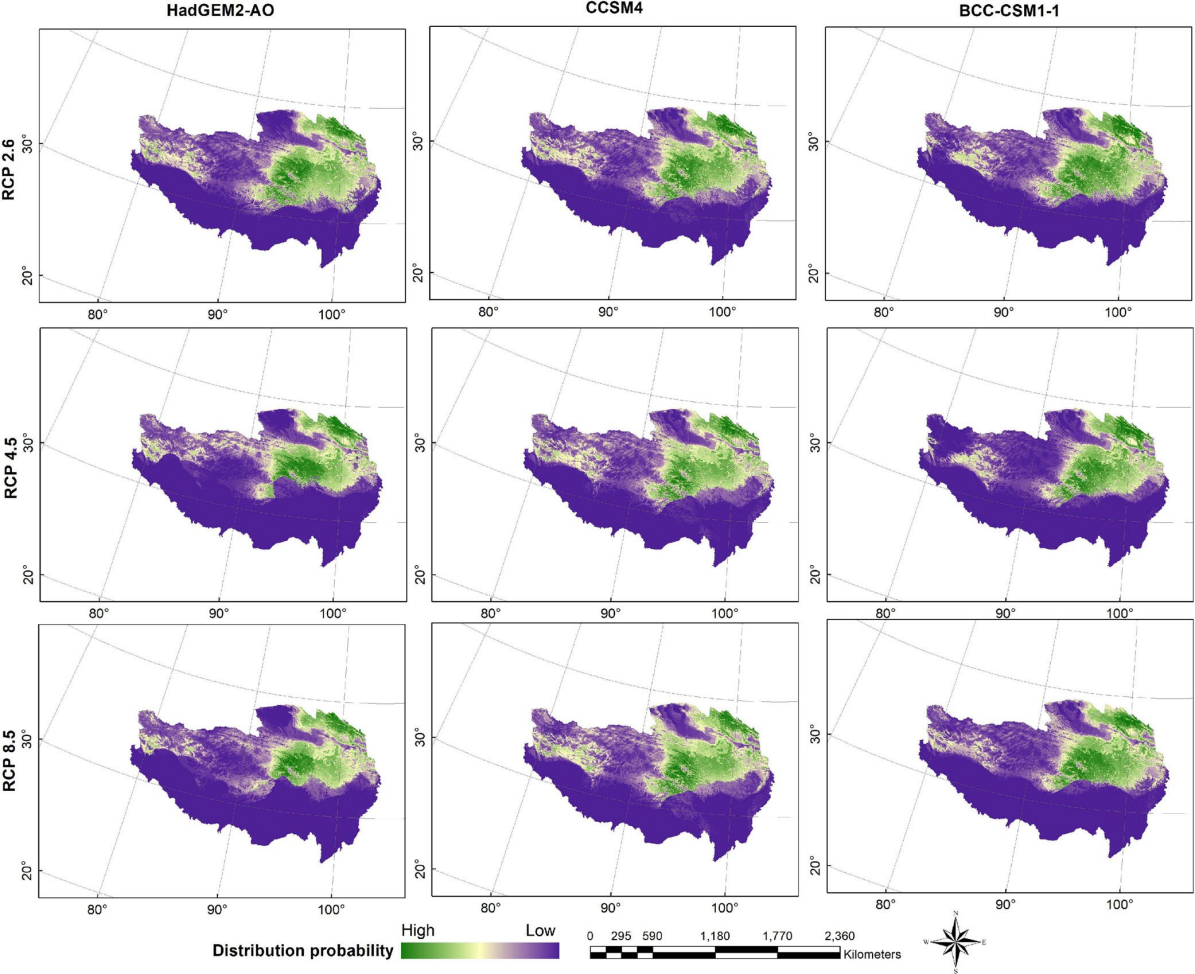
The distribution probability of snow leopard ( *Panthera uncia* ) under future (scenarios of RCP 2.6, RCP 4.5, and RCP 8.5 in the 2070s) climate and land use change scenarios on the Qinghai‐Tibet Plateau.

**FIGURE 6:**
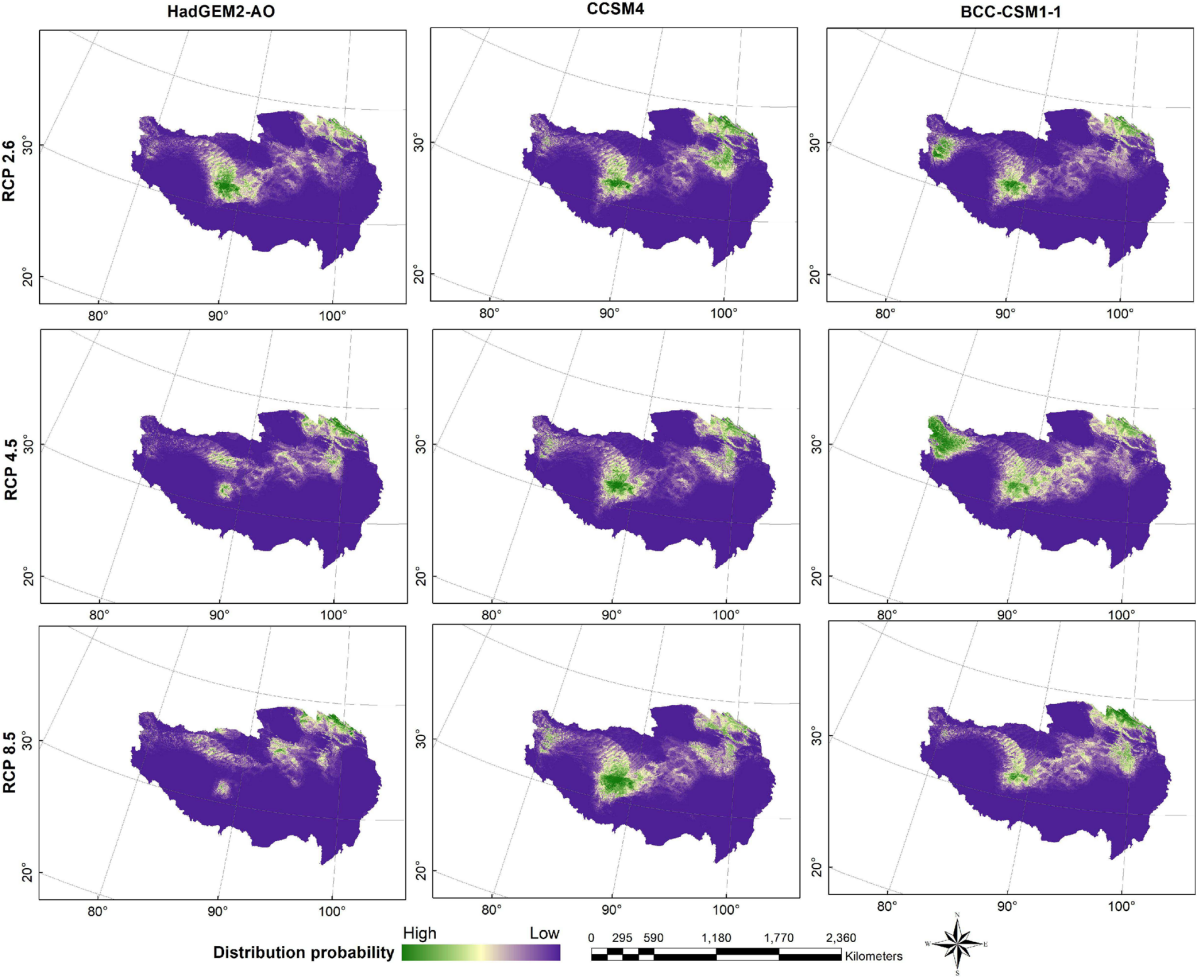
The distribution probability of blue sheep ( *Pseudois nayaur* ) under future (scenarios of RCP 2.6, RCP 4.5, and RCP 8.5 in the 2070s) climate and land use change scenarios on the Qinghai‐Tibet Plateau.

**FIGURE 7:**
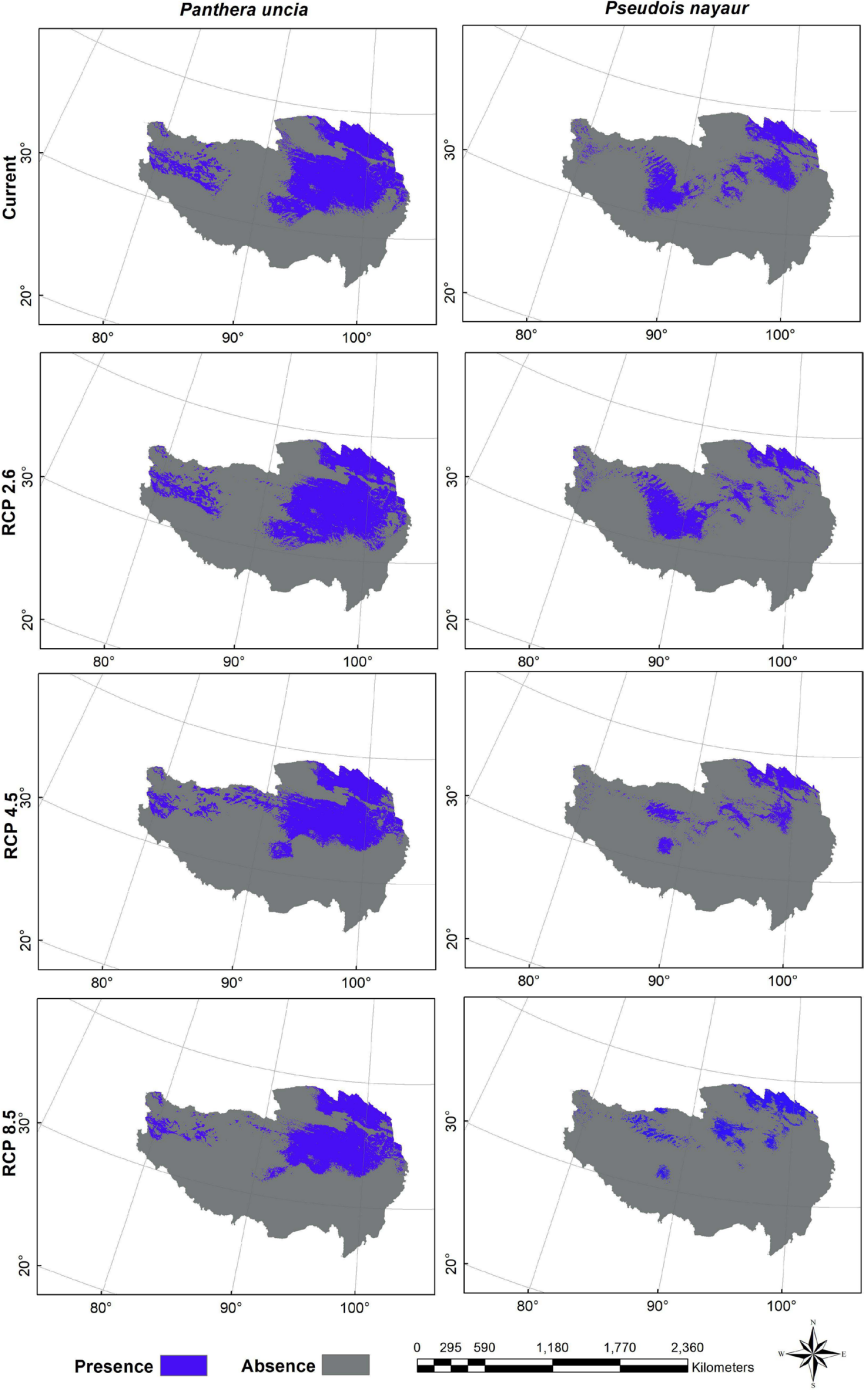
The binary distribution maps of snow leopard ( *Panthera uncia* ) and blue sheep ( *Pseudois nayaur* ) under the current and future (scenarios of RCP 2.6, RCP 4.5, and RCP 8.5 in the 2070s) climate and land use change scenarios on the Qinghai‐Tibet Plateau. Future distributions are the intersection area of the three GCMs (HadGEM2‐AO, CCSM4, and BCC‐CSM1‐1).

Figures 4, 5, 6 show the probability distributions of snow leopards and blue sheep under current and future climate scenarios and land use changes. The average MTSPS thresholds for snow leopards and blue sheep were 0.3091 and 0.1989, respectively. Binary distribution maps for both species were generated based on these MTSPS values (Figure 7). Under current and future climate scenarios and land use changes, Qinghai province held the largest proportion of potential distribution areas for snow leopards and blue sheep (Table 2, Figure 7).

**TABLE 2:** The potential distribution area of snow leopard ( *Panthera uncia* ) and its primary prey, blue sheep ( *Pseudois nayaur* ), under current and future climate scenarios and land use changes on the Qinghai‐Tibet Plateau.

Species	Province	Current		RCP 2.6		RCP 4.5		RCP 8.5	
		Area (km^2^)	Proportion (%)	Area (km^2^)	Proportion (%)	Area (km^2^)	Proportion (%)	Area (km^2^)	Proportion (%)
*Panthera uncia*	Qinghai	686982.03	69.56	659422.97	65.28	657006.55	76.42	616445.89	81.43
	Xizang	195612.05	19.81	222244.27	22.00	137168.69	15.96	72592.72	9.59
	Xinjiang	5142.43	0.52	4634.24	0.46	4559.88	0.53	5454.18	0.72
	Sichuan	63218.35	6.40	89258.84	8.84	28960.50	3.37	30972.34	4.09
	Gansu	36724.03	3.72	34613.30	3.43	32016.08	3.72	31598.22	4.17
*Pseudois nayaur*	Qinghai	296409.24	62.27	218765.71	45.94	223071.84	76.06	185189.76	77.44
	Xizang	174544.91	36.67	252106.98	52.94	69066.73	23.55	52795.98	22.08
	Xinjiang	3927.19	0.83	4489.65	0.94	838.28	0.29	847.87	0.35
	Sichuan	168.79	0.04	483.46	0.10	28.83	0.01	0.34	0.00
	Gansu	928.33	0.20	381.47	0.08	287.61	0.10	298.17	0.12

The current habitat of the snow leopard spanned 987,678.88 km^2^. Under the RCP 2.6 scenario, this habitat increased slightly to 1,010,173.61 km^2^, indicating a potential expansion under low‐emission conditions. However, under the RCP 4.5 and RCP 8.5 scenarios, the habitat was projected to decrease to 859,711.69 km^2^ and 757,063.34 km^2^, respectively, reflecting a loss of 13.0% and 23.4% compared to the current habitat. These reductions highlighted the species' vulnerability to climate change, particularly under higher emissions scenarios.

The current habitat of the blue sheep was 475,978.47 km^2^. Under the RCP 2.6 scenario, the habitat remained relatively stable, increasing slightly to 476,227.27 km^2^. However, significant reductions were projected under the RCP 4.5 and RCP 8.5 scenarios, with habitats shrinking to 293,293.30 km^2^ and 239,132.12 km^2^, respectively. This represented a 38.3% and 49.7% decrease compared to the current habitat, indicating a severe loss of suitable habitat for the species in response to climate change under higher emissions scenarios.

**FIGURE 8:**
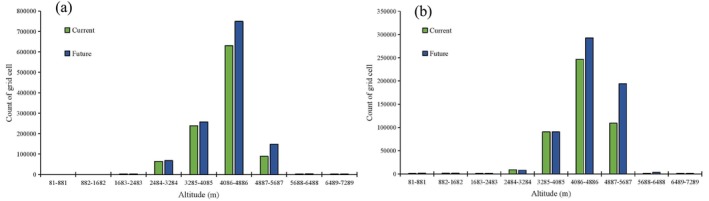
Altitudinal distribution patterns of (a) snow leopard ( *Panthera uncia* ) and (b) blue sheep ( *Pseudois nayaur* ) under current and future (the intersection map of three RCPs and three GCMs) climate and land use change scenarios on the Qinghai‐Tibet Plateau.

Under the influence of climate and land use changes, the distribution ranges of snow leopards and blue sheep shifted toward higher altitudes, with snow leopards exhibiting a greater magnitude of altitude change compared to blue sheep. Currently, 88,887 grid cells of habitat for snow leopards are located above 4887 m. By the 2070s, this number is projected to increase to 148,093 grid cells (Figure 8a). For blue sheep, 246,573 grid cells of habitat are currently distributed above 4086 m, which is expected to rise to 292,669 grid cells by the 2070s (Figure 8b). Presently and in the future, both snow leopards and blue sheep exhibit similar altitudinal ranges, predominantly found between 4086 m and 4886 m (Figure 8a,b).

### 3.4 Three Habitat Scenarios

**FIGURE 9:**
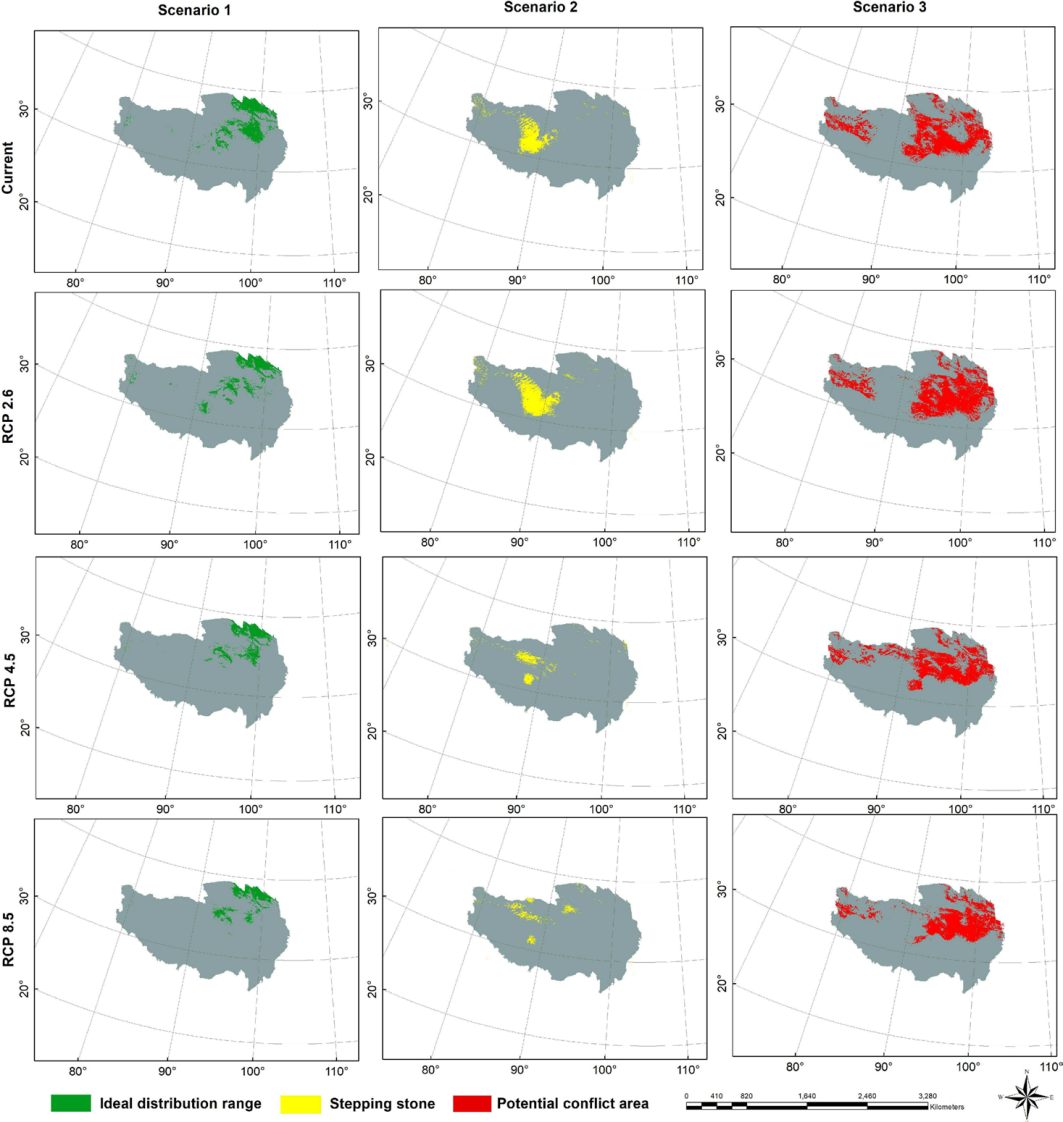
The three habitat scenarios (S1: Ideal distribution range; S2: Stepping stone; S3: Potential conflict area) of snow leopard ( *Panthera uncia* ) on the Qinghai‐Tibet Plateau.

In the current scenario, the areas of the ideal distribution range (S1), stepping stone (S2), and potential conflict area (S3) for snow leopards were 196,236.47 km^2^, 126,244.77 km^2^, and 462,501.56 km^2^, respectively. Significant changes in the area of different habitat scenarios occurred with the shift in climate change scenarios. Under the RCP 2.6 scenario, the area of the ideal distribution range (S1) slightly decreased to 147,741.36 km^2^, while the area of the stepping stone (S2) increased to 180,300.25 km^2^, and the potential conflict area (S3) expanded to 619,604.43 km^2^. Under higher RCP 4.5 and RCP 8.5 scenarios, the area of the ideal distribution range (S1) continued to decrease, whereas the areas of the stepping stone (S2) and potential conflict area (S3) exhibited different trends, reflecting the complex impacts of climate change and human activities on snow leopard habitats (Figure 9; Table 3).

**TABLE 3:** The statistics of three habitat scenarios (S1: Ideal distribution range; S2: Stepping stone; S3: Potential conflict area) of snow leopard ( *Panthera uncia* ) on the Qinghai‐Tibet Plateau.

	S1 (km^2^)	S2 (km^2^)	S3 (km^2^)
Current	196236.47	126244.77	462501.56
RCP 2.6	147741.36	180300.25	619604.43
RCP 4.5	139251.44	55842.33	509473.19
RCP 8.5	10794.82	50581.29	409792.80

## 4 Discussion

The impact of climate change on species distribution is a critical topic in biological and ecological research (Dunbar 1998). As global warming intensifies, ecosystems worldwide are undergoing significant changes that affect the distribution and survival of many species (Xian et al. 2022; Zhang et al. 2019). While this relationship is well‐established, our study offers novel insights by simulating the snow leopard's habitat, incorporating both the species and its prey, using metagenomic analysis of its diet. Unlike traditional habitat models, we explore three distinct distribution scenarios: ideal distribution range (S1), stepping stone (S2), and potential conflict area (S3). These new scenarios provide a fresh perspective on habitat shifts and human‐wildlife conflict, offering valuable insights into the effects of climate change on snow leopard and prey species habitats.

Climate change alters vegetation distribution patterns, thereby indirectly impacting the distribution patterns of ungulates and their predators (McKelvey and Buotte 2018). As global climate warming intensifies, significant changes in vegetation types and coverage are occurring in many regions worldwide, directly influencing habitat selection and distribution of ungulate animals (Zhang et al. 2022). Firstly, climate change exacerbates local droughts or alters precipitation patterns, which can suppress or promote vegetation growth in different ecosystems such as grasslands, deserts, and forests. For instance, grassland areas may experience reduced grass cover and changes in grass species composition, directly affecting the food supply and survival conditions for wild herbivores. Furthermore, changes in vegetation distribution impact the migration patterns and seasonal activities of ungulate animals. Ungulates rely on specific types of vegetation for food, shelter, and breeding sites. When their accustomed vegetation types change, they may need to adjust their migration routes or long‐term habitat choices to adapt to new environmental conditions. Moreover, these vegetation changes also influence the distribution patterns of predatory animals (Walker et al. 2019). Based on this understanding, we simulated the distribution of snow leopards and their main prey, blue sheep, to explore their interactions and responses to environmental changes.

In light of these environmental changes, the dietary plasticity of apex predators, such as snow leopards, represents a critical factor in their capacity to adapt to climate change. Snow leopards exhibit notable dietary flexibility, adjusting their prey preferences in response to seasonal availability and scarcity (Koju et al. 2023). This adaptability allows snow leopards to survive during periods when larger prey species, such as blue sheep, are less abundant, with smaller mammals compensating for the dietary gap during the winter months (Koju et al. 2023). Such dietary flexibility is vital for snow leopards as climate change drives fluctuations in prey populations due to shifts in ecosystems. Moreover, these dietary adjustments may influence interspecies interactions, as the presence or absence of certain prey species could have cascading effects throughout the entire food web (Lovari et al. 2013, 2024). Snow leopards' ability to switch between wild and domestic prey, including blue sheep and horses, further exemplifies their capacity to adapt to changing environmental conditions (Koju et al. 2023, 2024). This dietary plasticity may play a crucial role in maintaining population stability despite the challenges posed by climate change. However, the long‐term consequences of these dietary shifts on snow leopards health, reproduction, and their ecological interactions warrant further investigation. Consequently, integrating genetic tools and conducting long‐term ecological studies are essential for a comprehensive understanding of snow leopard dietary adaptability and its potential contribution to their conservation in the face of climate change.

In our study, we found that altitude and climate variables significantly contribute to the ecological models of snow leopards and blue sheep, indicating that climate change plays a critical role in regulating species' suitable habitats. With ongoing global warming, the climate patterns on the Qinghai‐Tibet Plateau are undergoing significant changes, profoundly impacting the distribution of snow leopards and blue sheep. Firstly, climate change is leading to a shift of suitable habitats for these species toward higher altitudes. For instance, our research on snow leopards showed a movement of their climatically suitable distribution toward higher altitudes and latitudes, possibly linked to the shrinking of suitable habitats at lower elevations due to rising temperatures. The distribution of suitable habitats for blue sheep also exhibited a similar trend, although responses may differ concerning more stringent seasonal precipitation changes. In addition, climate change exacerbates habitat instability and fragmentation for snow leopards and blue sheep. As temperature and precipitation patterns shift, formerly stable ecosystems are experiencing fragmentation and transformation, potentially leading to reduced species adaptability and population dispersal.

We observed significant reductions in the distribution ranges of snow leopards and blue sheep under different climate change scenarios, particularly under RCP 4.5 and RCP 8.5. Conversely, under the RCP 2.6 scenario, changes in habitat suitability were relatively minor. In the RCP 2.6 scenario, significant reductions in global greenhouse gas emissions are expected, stabilizing greenhouse gas concentrations at lower levels by the end of the century. Despite ongoing climate change, its impacts on high mountain ecosystems are comparatively minor in this scenario, with less drastic changes in habitat for snow leopards and blue sheep. In contrast, under RCP 4.5 and RCP 8.5, continued increases in global greenhouse gas emissions result in significant rises in greenhouse gas concentrations, amplifying the impacts of climate change on ecosystems. In these scenarios, rising temperatures and altered precipitation patterns in mountain regions could lead to considerable reductions in suitable habitats for snow leopards and blue sheep. These changes not only affect their habitat selection and migration patterns but may also profoundly impact their populations and survival. Therefore, our study underscores the potential impacts of global climate change on key species within high mountain ecosystems. Effective mitigation and adaptation measures are crucial in addressing future challenges posed by climate change to safeguard the habitats and ecological roles of these endangered species. Future research and conservation efforts should further explore trends in biodiversity under different climate scenarios, providing scientific foundations for sustainable natural resource management and conservation strategies.

Climate change may exacerbate human‐wildlife conflicts (HWCs) (Abrahms et al. 2023). As habitats in certain regions are lost and fragmented, wildlife is forced into more human‐populated areas in search of food and shelter, increasing the likelihood of direct human‐wildlife encounters and potentially sparking HWCs (Dai, Hacker et al. 2021). Our study explored the potential impacts of climate change on snow leopard habitat distribution and focuses on scenarios where human activities may intensify HWCs. Our findings revealed that with climate change, the area of S3 (potential conflict area) significantly expanded, while the area of S1 (Ideal distribution range) continued to decrease under RCP 4.5 and RCP 8.5 scenarios, reflecting the complexities of snow leopard habitats influenced by climate change and human activities. As climate change drives adaptive shifts in snow leopard habitats, they may move toward human settlements in search of food, further increasing direct interactions between humans and snow leopards and intensifying pressure on snow leopard habitats from human activities. Therefore, investigating the conflict dynamics between humans and snow leopards in the context of climate change is of significant scientific value.

While this study provides important insights into the potential impacts of climate change on snow leopard and blue sheep habitats, some limitations must be acknowledged. First, our study is based on models that rely on current data, which may not fully capture the complexities of climate change dynamics in these high‐altitude regions. Modeling habitat suitability under climate change scenarios often involves inherent uncertainties, particularly concerning future climate projections and the ability of species to adapt to rapid environmental shifts. In addition, while we focused on the major environmental variables such as temperature and precipitation, other ecological factors, such as the impact of disease, invasive species, and anthropogenic activities like poaching or infrastructure development, were not included in the models. These factors can significantly influence the distribution of species, and their exclusion may limit the precision of our predictions. Third, the spatial resolution of some climate models, particularly at the regional level, may not capture the fine‐scale habitat heterogeneity present in the Qinghai‐Tibet Plateau, which could lead to overgeneralized conclusions about habitat suitability. These limitations underscore the need for continued research and the development of more refined models to better predict the impacts of climate change on species and their habitats.

## 5 Conclusion and Recommendation

As global climate warming intensifies, the climate patterns on the Qinghai‐Tibet Plateau are undergoing significant changes, including rising temperatures and increased uncertainty in precipitation patterns. These changes directly impact the habitat and distribution patterns of snow leopards and blue sheep, placing them under increasing survival pressure. In our study, using metagenomic analysis, we confirmed that blue sheep are a primary food source for snow leopards. Subsequently, utilizing bioclimatic variables and LUCC data, combined with ecological modeling, we assessed the habitat suitability for snow leopards and blue sheep on the Qinghai‐Tibet Plateau and identified ideal distribution range, stepping stone, and potential conflict zones between humans and snow leopards based on three habitat scenarios. Our findings revealed that Qinghai province holds the largest proportion of potential distribution areas for snow leopards and blue sheep on the Qinghai‐Tibet Plateau. Under the RCP2.6 scenario, suitable habitats for snow leopards and blue sheep increased slightly. However, under the RCP 4.5 and RCP 8.5 scenarios, projections indicated reductions in the distribution ranges of snow leopards and blue sheep. Specifically, under RCP 4.5 and RCP 8.5, the distribution range of snow leopards decreased by 13.0% and 23.4%, respectively, while blue sheep decreased by 38.3% and 49.7%. With the influence of climate and land use changes, the distribution ranges of snow leopards and blue sheep were expected to shift to higher altitudes, with snow leopards showing a greater altitude shift compared to blue sheep. Furthermore, the area of snow leopard habitats under three habitat scenario hypotheses was projected to change by the 2070s, with S1 decreasing slightly while S2 and S3 showed different trends.

Biodiversity conservation is a critical issue in current global environmental management. To effectively protect snow leopards and their primary prey species, a series of comprehensive measures should be implemented, particularly focusing on habitat conservation under different scenarios. The composition of the snow leopard diet reveals that blue sheep, Himalayan marmot, and plateau pika constitute 78.8% of their diet, with blue sheep being the dominant prey species. Protecting these primary prey species, such as blue sheep, is vital for ensuring the long‐term survival of snow leopards. For habitats of S1, adjustments to existing protected area boundaries or the establishment of new protected areas should be considered based on S1 distribution. For example, expanding protected areas or creating new reserves could help preserve the integrity of critical habitats for species like the snow leopard and blue sheep. This approach not only ensures the integrity of existing habitats but also expands the coverage of protected areas, providing more habitat options and resource security for these species. For S2, it is recommended to establish ecological corridors for wildlife migration. In areas like the Qilian Mountains, creating corridors that link isolated habitats would facilitate natural migration and gene flow among species, such as the Tibetan antelope ( *Pantholops hodgsonii* ), reducing habitat fragmentation and improving species connectivity. For S3, a series of targeted measures to prevent HWCs should be implemented. For instance, in regions of Zhiduo county in Qinghai province, where conflicts with livestock are common, spatially differentiated management strategies, such as seasonal grazing bans or livestock relocation, could alleviate potential HWCs. These measures, along with community‐based approaches, can help minimize human‐wildlife conflicts while protecting the species' habitats.

Protecting biodiversity requires strong cross‐regional and cross‐administrative cooperation. Our research shows that under the RCP 4.5 and RCP 8.5 climate scenarios, suitable habitats for both snow leopards and blue sheep are projected to decrease, while these habitats are expected to expand under the RCP 2.6 scenario, contributing positively to biodiversity conservation. Strengthening habitat protection for these species requires intensified international collaboration and proactive measures to mitigate global carbon emissions. Reducing greenhouse gases, especially through low‐emission pathways, can effectively alleviate the negative impacts of climate change on their habitats, ensuring their long‐term survival and reproduction. Achieving this requires coordinated global actions, including policy development, technological innovation, and societal engagement to promote sustainable development and maintain ecosystem integrity. Additionally, establishing cross‐regional conservation networks is essential to protect migration corridors and habitats, mitigating human‐induced pressures. Advancing scientific research and monitoring is crucial to assess the long‐term effects of climate change on biodiversity and ecosystem functions, providing a scientific foundation for adaptive conservation strategies. This includes continuous monitoring of species populations, habitat conditions, and ecological trends, as well as evaluating the effectiveness of adaptive conservation interventions. Finally, fostering community education and engagement is vital to raise local awareness of wildlife conservation, encouraging active participation in the restoration and sustainable development of the Qinghai‐Tibet Plateau ecosystem.

## Author Contributions

**Yu Zhang:** funding acquisition (lead), writing – original draft (lead). **Yunchuan Dai:** writing – review and editing (equal). **Jia Li:** investigation (equal). **Wei Cong:** investigation (equal). **Yuguang Zhang:** writing – review and editing (equal). **Xiuqing Nie:** investigation (equal). **Qiong Wu:** writing – review and editing (equal). **Yadong Xue:** conceptualization (lead), funding acquisition (lead), writing – review and editing (equal).

## Conflicts of Interest

The authors declare no conflicts of interest.

## Data Availability

The original contributions presented in the study are included in this paper; further inquiries can be directed to the corresponding author.
